# Irisin Alleviates Cognitive Impairment by Inhibiting AhR/NF-*κ*B-NLRP3-Mediated Pyroptosis of Hippocampal Neurons in Chronic Kidney Disease

**DOI:** 10.1155/mi/2662362

**Published:** 2024-12-11

**Authors:** Jialing Zhang, Xingtong Dong, Qi Pang, Aihua Zhang

**Affiliations:** ^1^Department of Nephrology, Xuanwu Hospital, Capital Medical University, Beijing, China; ^2^National Clinical Research Center for Geriatric Disorders, Xuanwu Hospital, Capital Medical University, Beijing, China

**Keywords:** chronic kidney disease, cognition function, irisin, NLRP3, pyroptosis

## Abstract

**Introduction:** Cognitive impairment is a vital complication of chronic kidney disease (CKD). The effect of irisin on CKD-induced cognitive impairment remains unclear. In the present study, we aimed to investigate the role of Irisin in mitigating cognitive impairment and explore the underlying mechanisms in CKD.

**Methods:** A CKD mice model was established by adenine. Cognitive function was assessed via the novel object recognition (NOR). Interleukin-1*β* (IL-1*β*) levels were measured by enzyme-linked immunosorbent assay (ELISA), while pyroptosis-related protein expression was analyzed using western blotting.

**Results:** Our data showed an upregulation of cell pyroptosis in hippocampus tissues of CKD mice, accompanied by significant cognitive impairment. Pyroptosis and cognitive impairment was both improved by Irisin treatment in vivo. Additionally, irisin markedly downregulated pyroptosis levels through aryl hydrocarbon receptor (AhR)/NF-*κ*B p65 signaling in HT-22 cells pretreated with indoxyl sulfate (IS). In vitro experiments further confirmed that pyroptosis was inhibited by AhR and NF-*κ*B p65 inhibitors.

**Conclusions:** We first demonstrated that irisin alleviated cognitive impairment by inhibiting AhR/NF-*κ*B-NLRP3-mediated pyroptosis of hippocampal neurons in CKD. Overall, irisin may have the potential to serve as a critical antipyroptotic agent for improving CKD-induced cognitive impairment.

## 1. Introduction

Chronic kidney disease (CKD) is a major health concern worldwide. Cognitive impairment is a common comorbidity in CKD, mainly manifested as a deficit in one or more key brain functions, such as memory, learning, and decision-making. The prevalence of cognitive impairment in CKD is high, ranging from 16% to 87% [1, 2]. Additionally, a higher risk of cognitive impairment is significantly associated with the decline of residual renal function [3]. Cognitive dysfunction increased the burden of individuals with CKD. Emerging evidence showed that the decline in cognitive function is associated with an increasing risk of cardiovascular events and mortality, a poor quality of life, a worse ability to perform instrumental activities of daily living, and a large cost for families in CKD patients [4–7]. The pathophysiology of CKD-related cognitive impairment is multifactorial, involving vascular damage, uremic metabolites, inflammation, and oxidative stress [8]. However, there is currently insufficient evidence to establish specific strategies to improve cognitive performance in CKD patients.

Pyroptosis, a form of programmed cell death, plays a crucial role in multiple neurodegenerative disorders [9]. The pyroptotic pathway is associated with cognitive deficits in conditions such as inhaled anesthetic isoflurane [10], lysophosphatidylcholine-induced demyelination [11], and sepsis-associated encephalopathy [12]. However, whether the NLRP3/caspase-1 pyroptotic pathway contributes to CKD-induced cognitive dysfunction have not been reported.

Uremic toxins, such as indoxyl sulfate (IS), accumulates due to a gradual loss of kidney function, leading to a series of pathologies, including cerebrovascular disease, cardiovascular disease, anxiety, and cognitive impairment [13–16]. The aryl hydrocarbon receptor (AhR) is a ligand-regulated transcription factor, involved in various signaling pathways [17], including inflammation, immune system, and developmental processes of organs [18–22]. Indole compounds, produced of tryptophan metabolites, are potent endogenous ligands of AhR [23]. AhR signaling is implicated in age-related degenerative processes [24, 25] and CKD-related cognitive impairment [26, 27]. Compelling evidence showed that AhR factor co-operates with the NF-*κ*B signaling system [28], which involved in immune responses.

Irisin, a hormone-like molecules from skeletal muscle, is cleaved from fibronectin type III domain-containing protein 5 (FNDC5) and released into circulation [29]. Irisin could induce adipose tissue browning [29], improve cortical bone mineral density [30], and modulate brain function [31, 32]. FNDC5/irisin has been detected in the hippocampus to affect synaptic plasticity in Alzheimer's disease [33]. Circulating irisin is relatively lower in patients with CKD [34], suggesting a possible link between Irisin and cognitive impairment in CKD. However, the roles and mechanisms of irisin are unclear in CKD.

Therefore, this study hypothesizes and verified that irisin could inhibit pyroptosis by regulating AhR and NF-*κ*B pathway to improve CKD-associated cognitive dysfunction.

## 2. Materials and Methods

### 2.1. Antibodies and Reagents

The following antibodies were: NLRP3 (rabbit; ab263899, Abcam), AhR (rabbit; 28727-1-AP, Proteintech), N-GSDMD (rabbit; #10137, Cell signaling technology), cleaved caspase-1 (rabbit; #89332, Cell signaling technology), NF-*κ*B p65 (rabbit; #8242, Cell signaling technology), NF-*κ*B phospho-p65 (rabbit; #3033, Cell signaling technology), and anti-*β*-actin (mouse; #SC47778, Santa Cruz Biotechnology). Additionally, IS was acquired from Sigma, and irisin was acquired from Phoenix Pharmaceuticals. Creatinine (Cr) and blood urea nitrogen (BUN) assay kit were obtained from Nanjing Jiancheng Bioengineering Institute. Interleukin-1*β* (IL-1*β*) enzyme-linked immunosorbent assay (ELISA) kit was bought from Beyotime Biotechnology, and irisin ELISA kit was bought from Invitrogen. CH223191 and JSH-23 were both obtained from MedChemExpress. Kits of Hoechst33342/PI stanning were provided by Beyotime Biotechnology.

### 2.2. Cell Culture and Treatment

The mouse hippocampal neuron HT-22 cell line was purchased from the National Infrastructure of Cell Line Resource (Beijing, China). Cells were cultured in high-glucose DMEM (GIBCO, USA) supplemented with 10% FBS (Gibco, Waltham, MA, USA) and 1% penicillin/streptomycin (Solarbio, Beijing, China) at 37°C in a 95% air and 5% CO_2_ incubator. In the IS-treated group, HT-22 cells were incubated with IS at different concentrations (0.25 and 0.5 mM) for 48 h, which could significantly decrease the viability of HT-22 cells [35]. In the irisin-treated group, HT-22 cells were cotreated with IS and irisin to detect the effect of irisin on pyroptosis in vitro. In accordance with the previous study, different Irisin concentrations (50 and 100 ng/ml) were applied for 48 h [36]. At the end of stimulation, the cells were then collected for subsequent analysis.

### 2.3. ELISA

The levels of IL-1*β* in the medium of HT-22 cells were determined using an ELISA kit according to the manufacturer's instructions. The optical density of each well was measured at wavelength of 450 nm. Data were calculated based on the standard curves.

### 2.4. Cell Pyroptosis Analysis

Cell pyroptosis in the various treatment groups was analyzed using the Hoechst 33342 and propidium iodide (PI) staining. HT-22 cells were incubated with a mixed solution of Hoechst 33342 and PI after designated treated and photographed with a fluorescence microscope.

### 2.5. Animal Groups and Experimental Design

The experimental procedures and the animal use protocols were approved by the Ethics Committee of Xuanwu Hospital, Capital Medical University (No: XW-20220622-1). The experiments adhered to the Guide for the Care and Use of Laboratory Animals published by National Institutes of Health.

**Figure 1:**
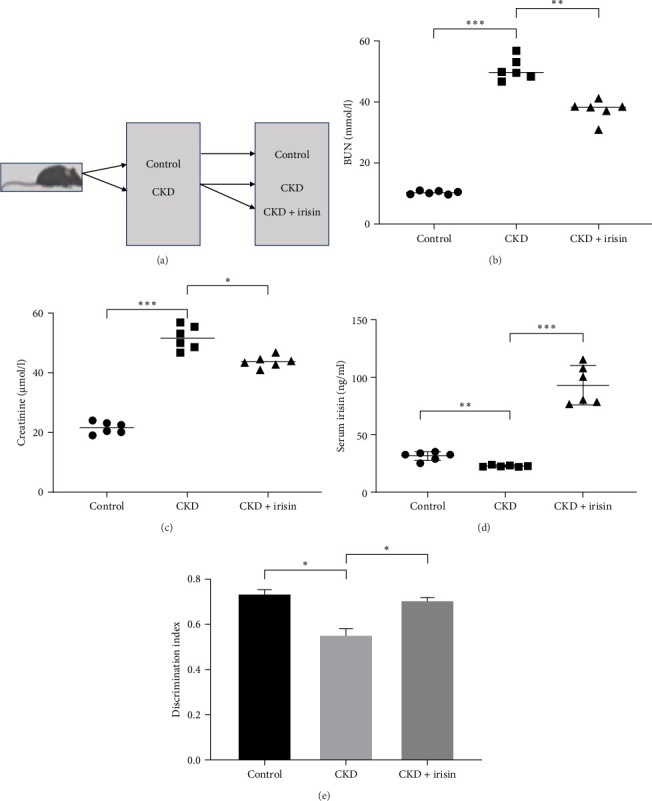
Irisin improved cognitive impairment in CKD mice. (A) Construction of the CKD mice model. (B) Serum levels of BUN. (C) Serum levels of Cr. (D) Serum levels of irisin. (E) The discrimination index in NOR test. *⁣*^*∗*^*p* < 0.05, *⁣*^*∗∗*^*p* < 0.01, *⁣*^*∗∗∗*^*p*  < 0.001. BUN, blood urea nitrogen; CKD, chronic kidney disease; Cr, creatinine; NOR, novel object recognition.

Male C57BL/6 mice aged 6 weeks (weighing 20–25 g) were purchased from the Model Animal Research Center of Nanjing University (Nanjing, China). The mice were housed at 22°C with a 12-h of light/dark cycle and free access to water and food. A CKD model was induced using an established adenine diet (0.2% adenine) for 4 weeks [37]. Mice were randomly divided into three groups: control, CKD, and CKD + irisin. In the control group, mice were fed with normal diet. The CKD group was fed the 0.2% adenine-containing diet for 4 weeks. In addition, to investigate the role of irisin, irisin (2 µg in 100 µl of normal saline per mouse; twice a week) was intravenously treated for 6–8 weeks [38]. Mice in the control and CKD groups received an equivalent volume of normal saline in the same manner. Finally, six mice survived in each group. The flowchart of the animal experiments was shown in Figure 1A.

### 2.6. Novel Object Recognition (NOR) Assay

The cognitive functions were assessed for all groups. NOR test is a 3-day protocol to measure the short- and long-term spatial memory associated with the hippocampus [39, 40]. The assay comprised three phases: habituation, familiarization, and testing [41]. During first day of NOR test, the experimental mice were placed in a 30 cm × 30 cm × 30 cm for 15 min adaptation. On day two, mice were placed individually in the same cage with two novel objects placed at opposite corners and allowed to explore freely for 10 min. On day three, one object was replaced with a new object and explored for 10 min. The time spent with each object was recorded. A discrimination index was calculated by dividing the time spent exploring the novel object by the total time spent exploring both objects to evaluate memory.

### 2.7. Biochemical and Cytokine Analysis

At the end of the animal experiment, mice were sacrificed by intraperitoneal injection of an overdose of pentobarbital sodium (1 mg/kg). After the mice were sacrificed, blood sample was centrifuged at 800 rpm for 10 min at 4°C. The supernatant serum was aliquoted and stored at −80°C for analysis of IL-1*β* and irisin using commercial kits. The levels of serum Cr and BUN were measured according to the manufacturer's protocol, respectively.

### 2.8. Western Blot

Total protein was extracted from hippocampal tissue or cultured HT-22 cells using RIPA lysis buffer (Beyotime, China) containing protease inhibitor and phosphatase inhibitor (Beyotime, China). Protein concentrations were determined using a BCA Kit (Beyotime, China). Equal amounts of protein were separated by 12.5% sodium dodecyl sulfate-polyacrylamide gel electrophoresis and then transferred to a PVDF membrane. The membrane was then blocked with 5% nonfat dry milk and incubated overnight at 4°C with primary antibodies against NLRP3 (1:1000), AhR (1:2000), N-GSDMD (1:1000), cleaved caspase-1 (1:1000), NF-*κ*B p65 (1:1000), and NF-*κ*B phospho-p65 (1:1000). After washed with TBST for three times, membranes were incubated with secondary antibody (a horseradish peroxidase (HRP)-conjugated goat anti-mouse or anti-rabbit IgG) for 1 h at room temperature. Electrogenerated chemiluminescence kits were applied to detect the signal, and the relative intensity was analyzed by Image J software (NIH, USA).

### 2.9. Statistical Analysis

GraphPad Prism 8.0 software was adopted for statistical analysis. Data are expressed as mean ± SEM. Multiple comparisons between experimental groups were analyzed using one-way analysis of variance (ANOVA). Statistical significance was set as a *p* value < 0.05.

## 3. Results

### 3.1. Irisin Treatment Reverted CKD-Associated Cognitive Impairment

To investigate the effect of irisin on CKD-induced cognitive impairment, a CKD model was established by an adenine diet for 4 weeks. The model was validated by measuring the serum levels of Cr and BUN (Figure 1B,C), which were significantly higher in CKD mice compared to controls. In addition, serum levels of irisin decreased in CKD mice compared to controls (Figure 1D).

The NOR test was performed to assess cognitive function. The discrimination index was reduced in CKD mice but tended to improve with Irisin treatment (Figure 1E). These results indicated that irisin could protect against cognitive impairment in CKD.

### 3.2. IS Could Increase NLRP3-Mediated Pyroptosis In Vitro and In Vivo

**Figure 2:**
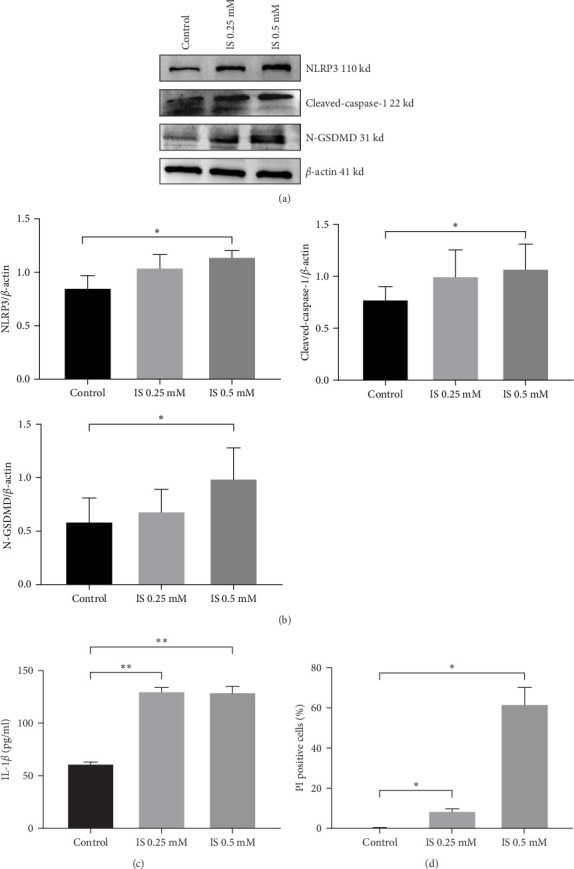
IS exposure induces pyroptosis in HT-22. (A, B) Protein levels of NLRP3, cleaved-caspase-1, and N-GSDMD were determined by western blotting. (C) IL-1*β* levels in HT-22 culture supernatants measured by ELISA. (D) Percentages of pyroptosis using Hoechst 33342/PI staining. Data are expressed as mean ± SEM (*n* = 3 for each group). *⁣*^*∗*^*p* < 0.05, *⁣*^*∗∗*^*p* < 0.01, and *⁣*^*∗∗∗*^*p*  < 0.001. ELISA, enzyme-linked immunosorbent assay; IL-1*β*, interleukin-1*β*; IS, indoxyl sulphate; PI, propidium iodide.

**Figure 3:**
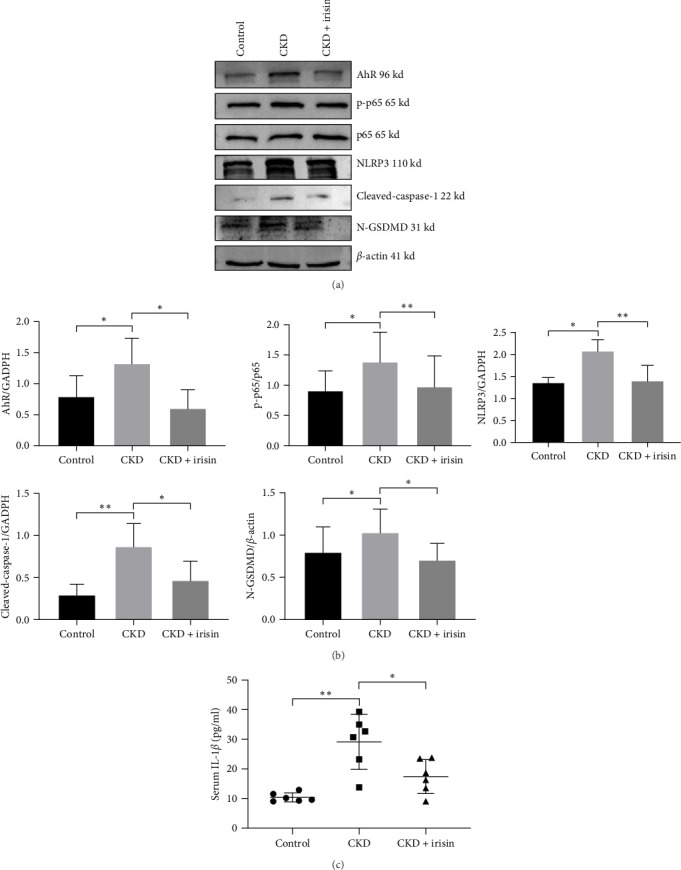
Irisin alleviates pyroptosis in CKD mice. (A, B) Protein levels of AhR, NF-*κ*B p65, NF-*κ*B p-p65, NLRP3, cleaved-caspase-1, and N-GSDMD were determined by western blotting. (C) Serum IL-1*β* levels in CKD mice measured by ELISA. Data are expressed as mean ± SEM (*n* = 3 for each group). *⁣*^*∗*^*p* < 0.05, *⁣*^*∗∗*^*p* < 0.01. AhR, aryl hydrocarbon receptor; CKD, chronic kidney disease; ELISA, enzyme-linked immunosorbent assay; IL-1*β*, interleukin-1*β*.

IS was associated with cognitive impairment in CKD [42]. In this research, IS treatment significantly increased the expression of NLRP3 and pyroptosis-related proteins in HT-22 cells by western blot (Figure 2A,B). ELISA was used to evaluate the level of neuroinflammation, and IL-1*β* level was significantly elevated in the IS-treated group (Figure 2C). As shown in the Figure 2D and Supporting information, Hoechst/PI staining showed a marked increase in PI-positive cells. To further clarify the effect of IS treatment on pyroptosis in vivo, the western blot results showed that CKD mice had a significantly higher expression levels of pyroptosis-related proteins in comparison to the control group (Figure 3A,B). Additionally, the concentrations of serum IL-1*β* in CKD mice were significantly higher (Figure 3C). Thus, our results suggested that IS (0.5 mM) could induce pyroptosis in CKD, and IS (0.5 mM) was determined in the subsequent experiments.

### 3.3. Irisin Attenuated NLRP3-Mediated Pyroptosis Induced by IS in Vitro and in Vivo

**Figure 4:**
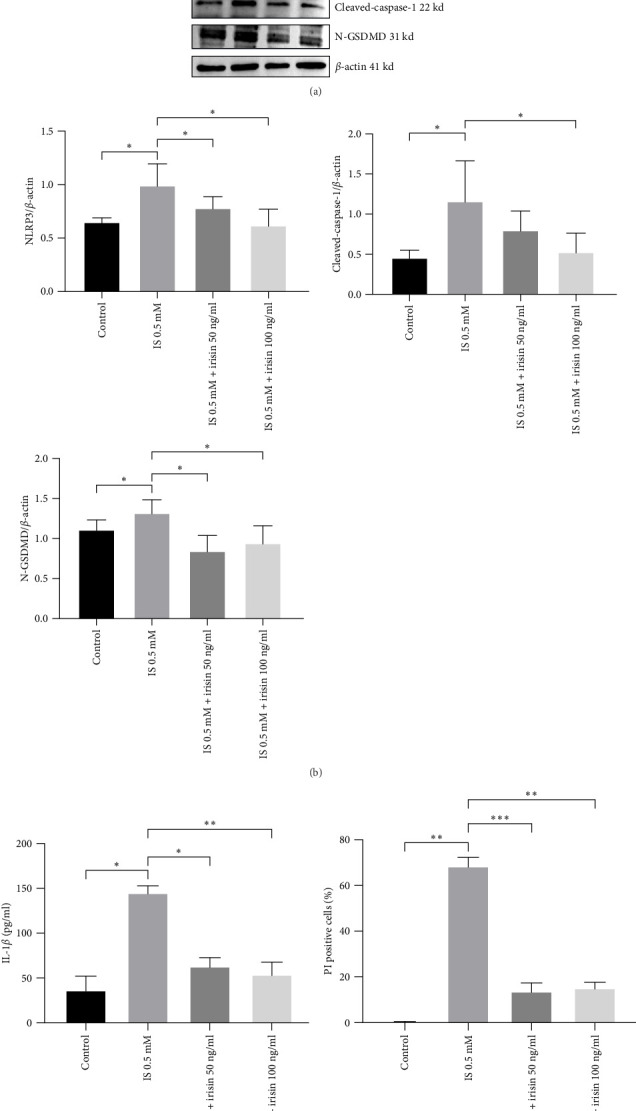
Irisin attenuates IS-induced pyroptosis in HT-22. (A, B) Protein levels of NLRP3, cleaved-caspase-1, and N-GSDMD were determined by western blotting. (C) IL-1*β* levels in HT-22 culture supernatants measured by ELISA. (D) Percentages of pyroptosis using Hoechst 33342/PI staining. Data are expressed as mean ± SEM (*n* = 3 for each group). *⁣*^*∗*^*p* < 0.05, *⁣*^*∗∗*^*p* < 0.01, and *⁣*^*∗∗∗*^*p*  < 0.001. ELISA, enzyme-linked immunosorbent assay; IL-1*β*, interleukin-1*β*; IS, indoxyl sulphate; PI, propidium iodide.

To verify the hypothesis that Irisin affects pyroptosis, HT-22 cells were treated with IS (0.5 mM) in the presence of Irisin (50 and 100 ng/ml). Consequently, western blot results revealed that IS treatment significantly increased protein levels of NLRP3, cleaved-caspase-1, and N-GSDMD. Additionally, cell pyroptosis was substantially reversed by the irisin treatment (Figure 4A,B). According to the ELISA results, irisin inhibited the secretion of IL-1*β* induced by IS treatment (Figure 4C). In addition, irisin reduced the proportion of pyroptotic cells (Figure 4D and Supporting information).

Consistent with the above results, we found that the expressions of NLRP3-mediated pyroptosis were also downregulated in the CKD mice treated with irisin (Figure 3A,B). Additionally, irisin significantly reduced the levels of Il-1*β* (Figure 3C). Collectively, these findings indicated that irisin could alleviate pyroptosis in hippocampus tissues.

### 3.4. Irisin Attenuated CKD-Associated Cognitive Impairment via Inhibiting the Activation of AhR/NF-*κ*B-Mediated Pyroptosis

**Figure 5:**
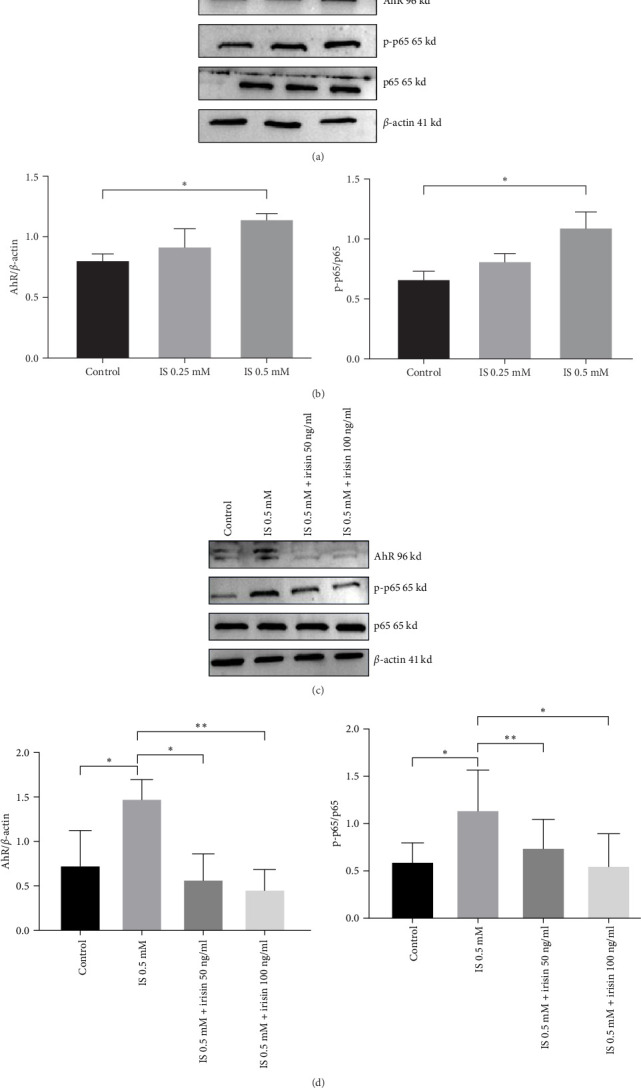
Irisin attenuates IS-induced pyroptosis via AhR/NF-*κ*B in HT-22. (A–D) Protein levels of AhR, NF-*κ*B p-65, and NF-*κ*B p-p65 were determined by western blotting. Data are expressed as mean ± SEM (*n* = 3 for each group). *⁣*^*∗*^*p* < 0.05, *⁣*^*∗∗*^*p* < 0.01, and *⁣*^*∗∗∗*^*p*  < 0.001. AhR, aryl hydrocarbon receptor; IS, indoxyl sulphate.

In this study, we observed increased AhR expression and phosphorylation of NF-*κ*B p65 in HT-22 cells following IS treatment (Figure 5A,B). To explore the mechanism behand irisin-mediated inhibition of pyroptosis. Western blot analysis showed that HT-22 cells pretreated with irisin reduced protein levels of AhR and NF-*κ*B p-p65 (Figure 5C,D). Similarly, CKD mice exhibited increased AhR and phosphorylation of NF-*κ*B p65, accompanied by a triggered pyroptosis (Figure 3A,B). In the CKD mice treated with irisin, a reduction of AhR and NF-*κ*B p-p65 was observed in the hippocampus.

**Figure 6:**
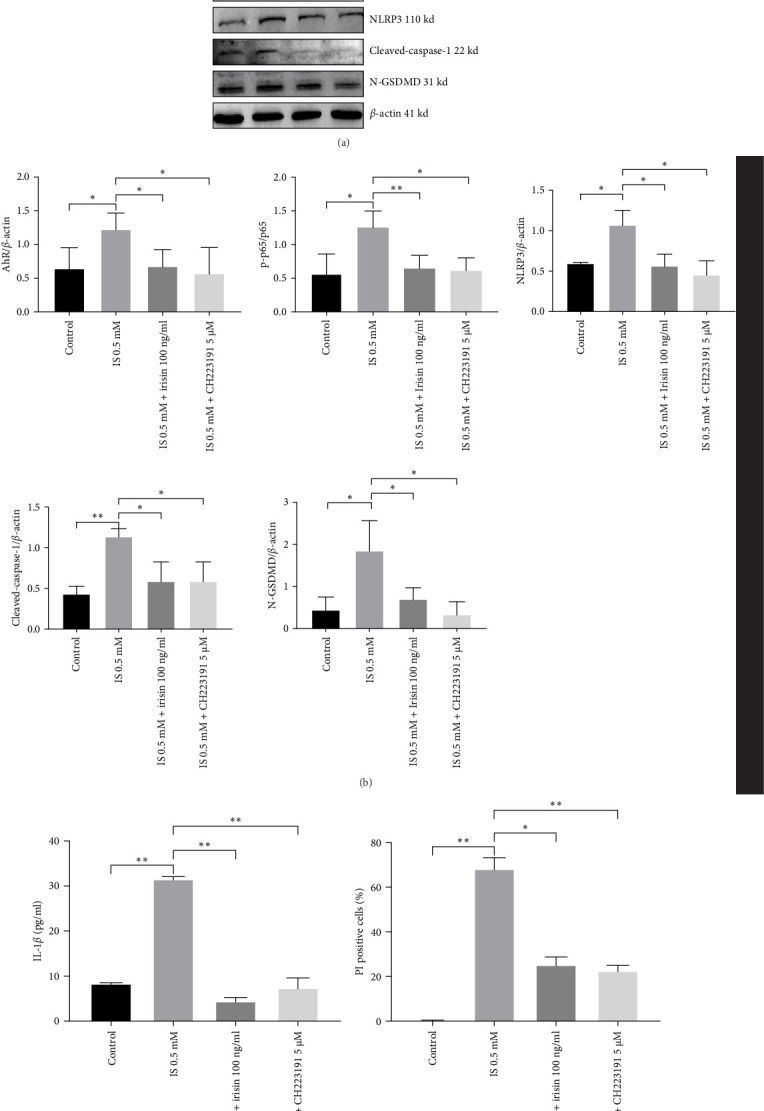
Inhibition of AhR attenuates IS-induced pyroptosis in HT-22. (A, B) Protein levels of AhR, NF-*κ*B p65, NF-*κ*B p-p65, NLRP3, cleaved-caspase-1, and N-GSDMD were determined by western blotting. (C) IL-1*β* levels in HT-22 culture supernatants measured by ELISA. (D) Percentages of pyroptosis using Hoechst 33342/PI staining. Data are expressed as mean ± SEM (*n* = 3 for each group). *⁣*^*∗*^*p* < 0.05, *⁣*^*∗∗*^*p* < 0.01, and *⁣*^*∗∗∗*^*p*  < 0.001. AhR, aryl hydrocarbon receptor; ELISA, enzyme-linked immunosorbent assay; IL-1*β*, interleukin-1*β*; IS, indoxyl sulphate; PI, propidium iodide.

**Figure 7:**
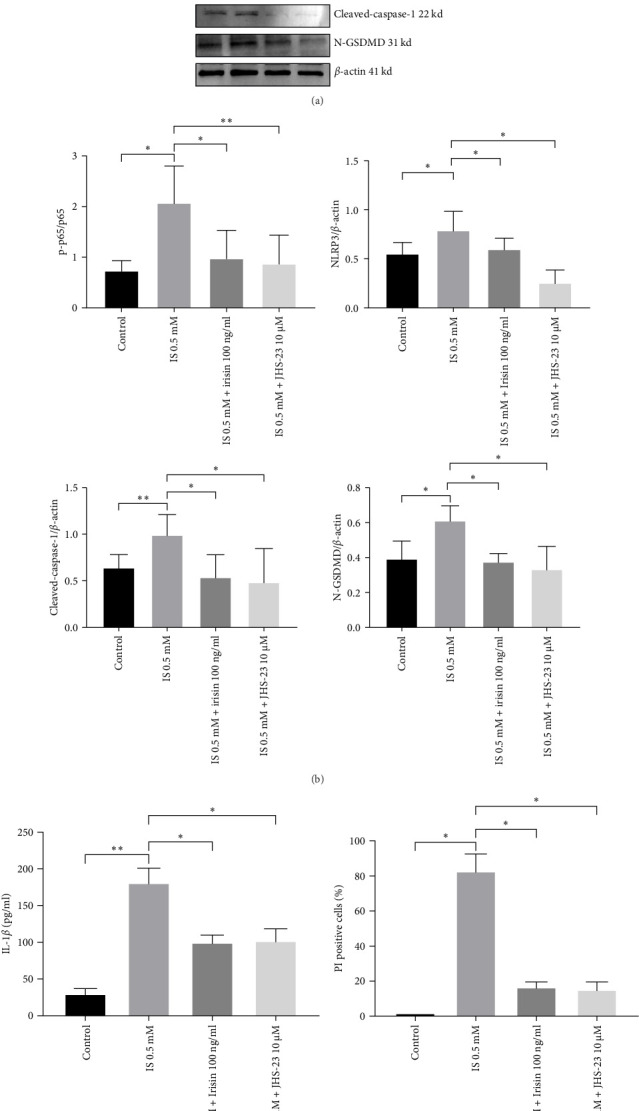
Inhibition of NF-*κ*B p-p65 attenuates IS-induced pyroptosis in HT-22. (A, B) Protein levels of NF-*κ*B p65, NF-*κ*B p-p65, NLRP3, cleaved-caspase-1, and N-GSDMD were determined by western blotting. (C) IL-1*β* levels in HT-22 culture supernatants measured by ELISA. (D) Percentages of pyroptosis using Hoechst 33342/PI staining. Data are expressed as mean ± SEM (*n* = 3 for each group). *⁣*^*∗*^*p* < 0.05, *⁣*^*∗∗*^*p* < 0.01, and *⁣*^*∗∗∗*^*p*  < 0.001. ELISA, enzyme-linked immunosorbent assay; IL-1*β*, interleukin-1*β*; IS, indoxyl sulphate; PI, propidium iodide.

To further validate our hypothesis, we individually used AhR inhibitor (CH223191) and NF-*κ*B p65 inhibitor (JSH-23) in the subsequent experiments. Both CH223191 and JSH-23 treatments reduced NLRP3, cleaved-caspase-1, and N-GSDMD levels (Figures 6A,B and 7A,B). Furthermore, the secretion of IL-1*β* was also lowered in comparison to the IS treatment (Figures 6C and 7C). Similarly, CH223191 and JSH-23 suppressed the percentage of PI-positive cells (Figures 6D and 7D and Supporting information). These data indicated that the activation of AhR/NF-*κ*B p65 pathway could amplify pyroptosis in CKD.

## 4. Discussion

In this study, we first demonstrated that NLRP3-mediated pyroptosis was closely related to the CKD-associated cognitive impairment. Furthermore, we provided evidence that irisin might improve cognitive function in CKD through inhibiting pyroptotic cell death and AhR/NF-*κ*B p65 signal pathway.

Neurological complications are common in CKD patients, contributing to poor quality of life and high morbidity and mortality [43]. The link between CKD and cognitive dysfunction is multifactorial, containing genetic factors, glymphatic system, vascular dysfunction, neuroinflammation, and uremic toxins [44]. The accumulation of uremic toxins, due to the gradual loss of kidney structure and function, could lead to neurotoxicity and blood–brain barrier injury [13]. IS, a protein-bound uremic toxin, could accumulate in the brainstem, cerebellum, and hippocampus, causing behavioral alterations in rats [45]. The increasing IS levels induced oxidative stress, free radicals, and inflammation and were independently associated with poor executive function in CKD patients [46]. However, the definite pathogenesis remains poorly understood.

Pyroptosis is a programmed cell death depending on the activation of caspase-1 in the canonical pathway. Hitherto, the NLPR3/caspase-1 mediated pyroptosis pathway has been implicated in cognitive impairment in cerebral ischemia/reperfusion injury, Alzheimer's disease and perioperative neurocognitive disorders [47–49]. Inhibition of NLRP3 inflammasome has been shown to reduce neuronal cell death [50]. However, the relationship between pyroptosis and cognitive impairment in CKD patients is inconclusive.

In this study, we found elevated levels of NLRP3, cleaved-caspase-1, and N-GSDMD in both the hippocampus of CKD mice and in HT-22 cells treated with IS. In addition, the performance of cognition behavior was impaired in CKD mice. Accumulating evidence demonstrated that hippocampus pyroptosis induced by sevoflurane, ammonia, and *βγ*-CAT contributed to cognition function [10, 51, 52]. Thus, we speculated that pyroptosis of hippocampus might participant in the CKD associated-neurocognitive impairment.

IS is known to be a ligand for AhR, activating its transcriptional activity [53, 54]. AhR is associated with cognitive dysfunction through oxidative stress [55], neuroinflammation [56], and impairing NO production [57]. IS treatment could increase inflammation via activating AhR/NF-*κ*B pathway in primary central nervous system cells, primary human valvular interstitial cells, and human macrophages [27, 58, 59]. NF-*κ*B is a crucial transcription factor in pyroptosis [60, 61]. The NF-*κ*B/NLRP3 axis is associated with diabetes-induced podocyte injury [62], contrast-induced acute kidney injury [63], and sepsis-induced acute kidney injury [64]. Importantly, our study verified that the expression of AhR and phosphorylation of NF-*κ*B were upregulated during IS treatment. The activation of AhR/NF-*κ*B signaling could induce pyroptosis in hippocampus and contribute to CKD-associated cognition impairment.

Irisin is a newly identified exercise hormone, and played a beneficial role in glucose homeostasis, insulin resistance, and blood pressure [65, 66]. The expression of irisin significantly decreased in CKD [67], with low levels linked to increases risks of abdominal aortic calcification in peritoneal dialysis patients [68] and mortality in hemodialysis patients [69]. In addition, a bridging role of exercise and neurological diseases was prompted. FNDC5 is expressed in hippocampus tissue [70], and irisin presented in the cerebrospinal fluid [71]. A protective effect of irisin against neurodegenerative diseases has been clarified. Irisin mediated a protective effect on Alzheimer's disease and Parkinson's disease by inhibiting neuroinflammation [72], regulating brain-derived neurotrophic factor [73], and synaptic plasticity [33]. However, the roles and mechanism of irisin in CKD-associated cognitive attenuation remains unclear.

In our current study, irisin significantly improved cognition function in CKD mice. Moreover, irisin decreased the levels of NLRP3, cleaved-caspase-1, and N-GSDMD, suggesting its role in suppressing pyroptosis in CKD. In addition, irisin significantly lowered the level of IL-1*β* induced by IS. Emerged evidence showed that Irisin could reduce pyroptosis in various diseases [74–76], although its underlying mechanisms are not fully elucidated. Our present study first demonstrated that Irisin could inhibit the activation of AhR and phosphorylation of NF-*κ*B in IS-treated HT-22 cells and the hippocampus of CKD mice. Additionally, inhibitor of AhR (CH223191) and NF-*κ*B (JSH-23) remarkably inhibited the expression of pyroptosis-related proteins and pyroptotic cell death in HT-22 cells. The protective effects of irisin are similar to those produced by CH223191 and JSH-23. Taken together, our findings indicated that Irisin may protect against hippocampal neurons pyroptosis through the AhR/NF-*κ*B signal pathway in CKD. Our results may provide novel inspiration for potential therapeutic approach to ameliorate cognition deficits by targeting the AhR/NF-*κ*B pathway.

There are some limitations to be noted. First, the mechanism of knocking down key molecules on the pyroptosis signaling are warranted to validate our results. Second, the utilization of antagonists in CKD mice models were absent. Future in vivo experiments using AHR knockout mice or antagonists could provide a better understanding of the roles of AhR/NF-*κ*B pathway.

## 5. Conclusion

The present study first prompted that irisin could attenuate CKD-associated cognitive impairment through inhibiting AhR/NF-*κ*B/NLRP3-mediated hippocampal neurons pyroptosis. Based on our results, irisin may provide a novel therapeutic strategy for addressing CKD-related cognitive deficits in the future.

## Data Availability

All data generated or analyzed during this study are available from the corresponding author on reasonable request.

## Supporting Information

Supporting Information Figure S1. Indoxyl sulfate exposure induced pyroptosis in HT-22 using Hoechst 33342/PI staining. Figure S2. Irisin attenuates indoxyl sulfate-induced pyroptosis in HT-22 using Hoechst 33342/PI staining. Figure S3. Inhibition of AhR attenuates indoxyl sulfate-induced pyroptosis in HT-22 using Hoechst 33342/PI staining. Figure S4. Inhibition of NF-*κ*B p-p65 attenuates indoxyl sulfate-induced pyroptosis in HT-22 using Hoechst 33342/PI staining.
