# Supplementary material for: Irisin Alleviates Cognitive Impairment by Inhibiting AhR/NF-κB-NLRP3-Mediated Pyroptosis of Hippocampal Neurons in Chronic Kidney Disease
Source: Mediators Inflamm. 2024 Dec 11;2024:2662362. doi: 10.1155/mi/2662362 (PMC11655147; doi:10.1155/mi/2662362)

Supplemental Figure 1 Indoxyl sulfate exposure induced pyroptosis in HT-22 using Hoechst 33342/PI staining.

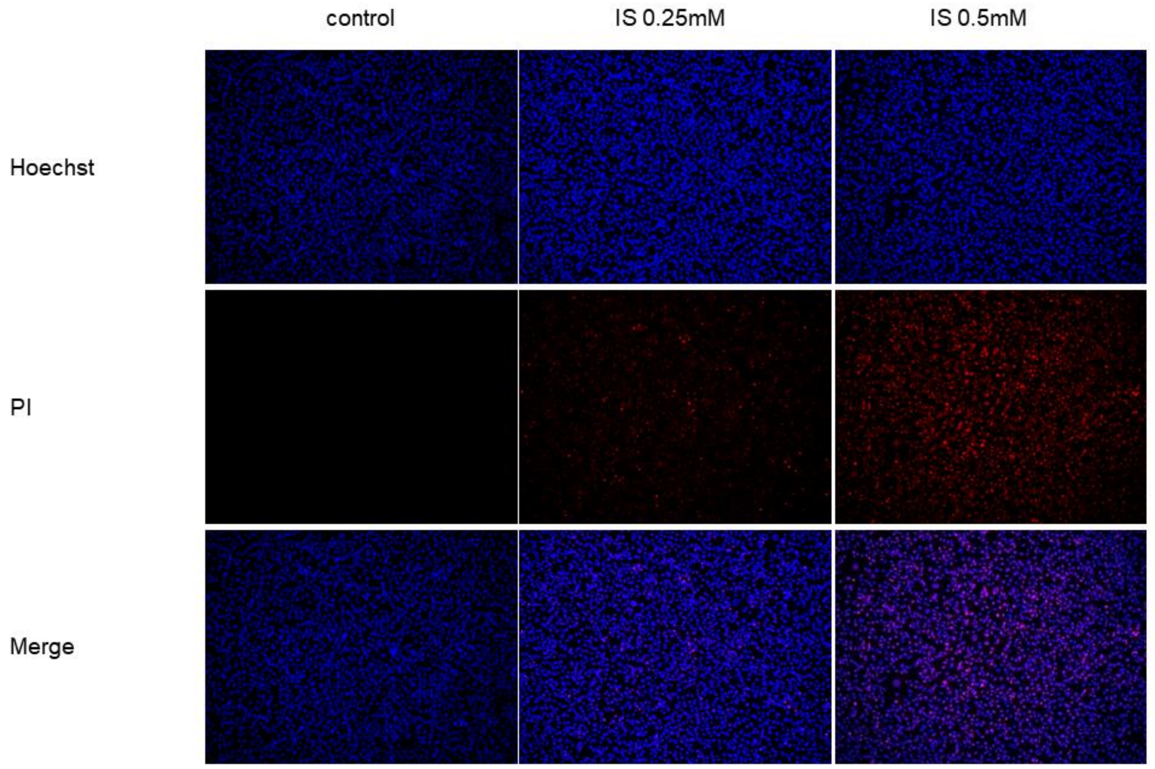

Supplemental Figure 2 Irisin attenuates indoxyl sulfate-induced pyroptosis in HT-22 using Hoechst 33342/PI staining.

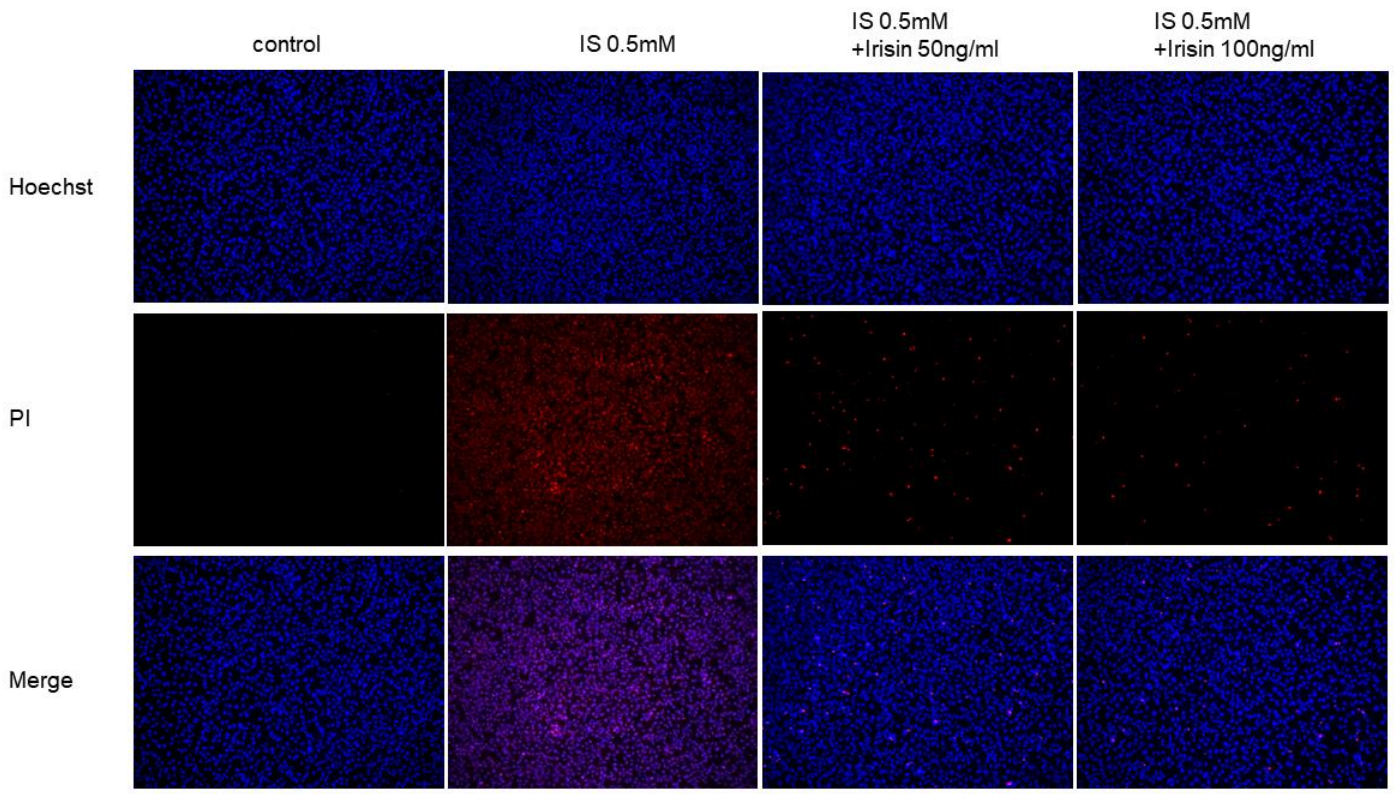

Supplemental Figure 3 Inhibition of AhR attenuates indoxyl sulfate-induced pyroptosis in HT-22 using Hoechst 33342/PI staining.

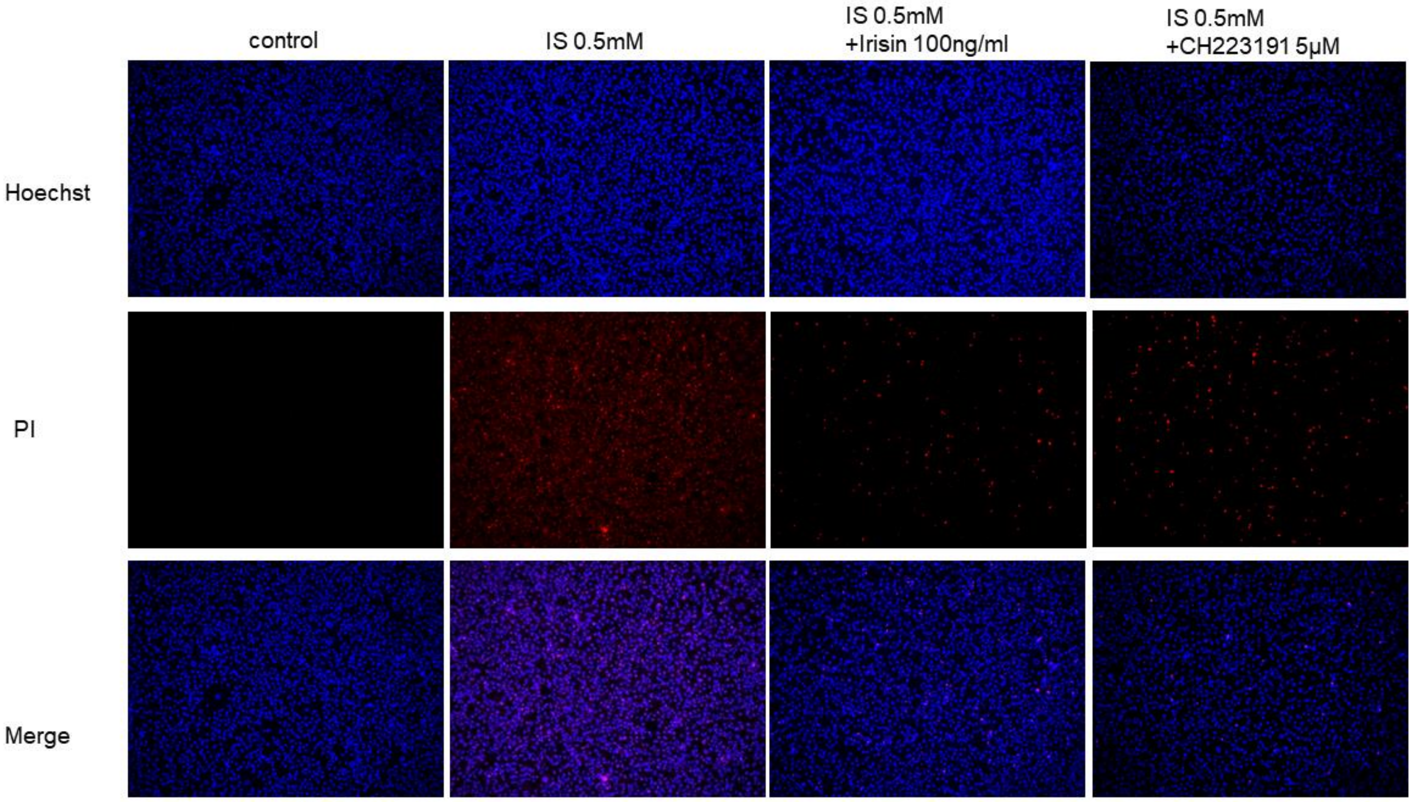

Supplemental Figure 4 Inhibition of NF-κB p-p65 attenuates indoxyl sulfate-induced pyroptosis in HT-22 using Hoechst 33342/PI staining.

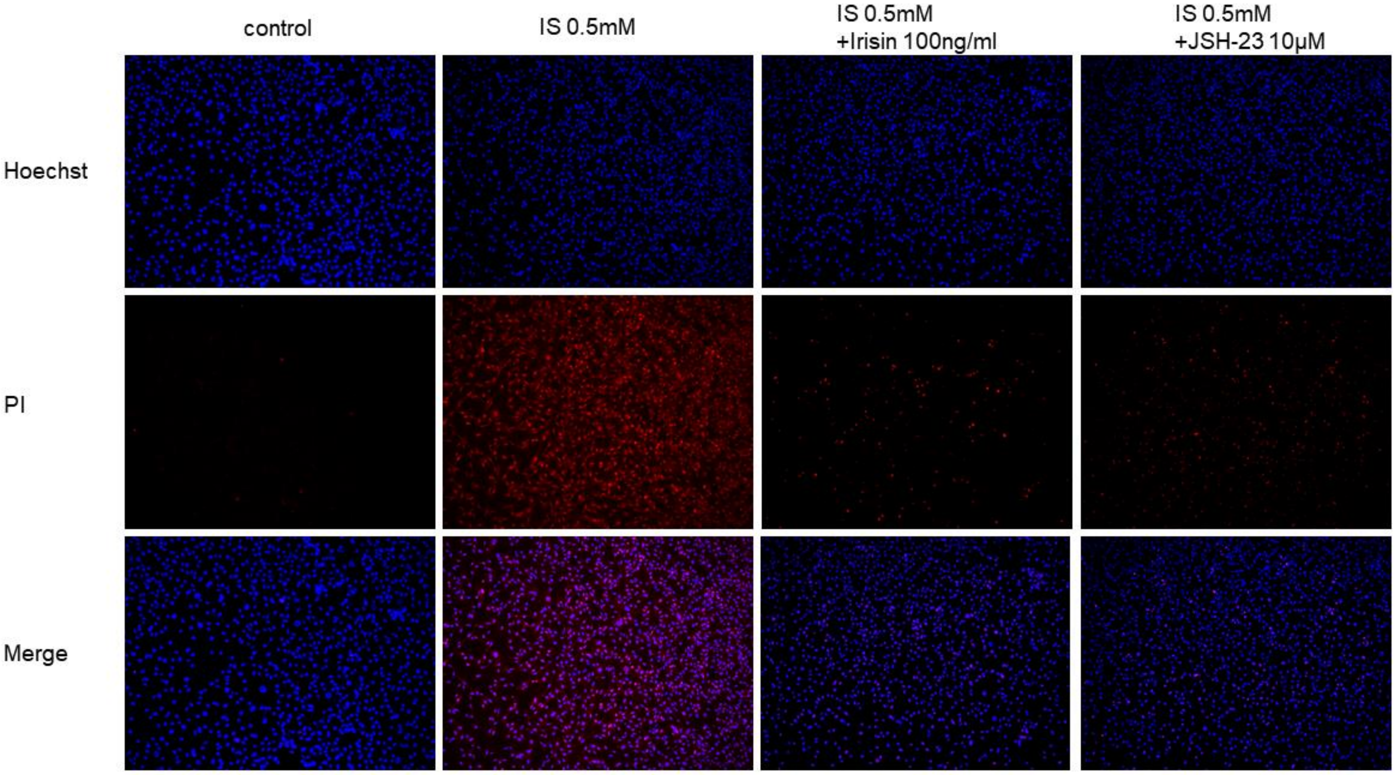

Supplement: Supporting Information — Figure S1. Indoxyl sulfate exposure induced pyroptosis in HT-22 using Hoechst 33342/PI staining. Figure S2. Irisin attenuates indoxyl sulfate-induced pyroptosis in HT-22 using Hoechst 33342/PI staining. Figure S3. Inhibition of AhR attenuates indoxyl sulfate-induced pyroptosis in HT-22 using Hoechst 33342/PI staining. Figure S4. Inhibition of NF-κB p-p65 attenuates indoxyl sulfate-induced pyroptosis in HT-22 using Hoechst 33342/PI staining. [file 2662362.f1.pdf]
